# Religion and Faith Perception in a Pandemic of COVID-19

**DOI:** 10.1007/s10943-020-01088-3

**Published:** 2020-10-12

**Authors:** Oliwia Kowalczyk, Krzysztof Roszkowski, Xavier Montane, Wojciech Pawliszak, Bartosz Tylkowski, Anna Bajek

**Affiliations:** 1 Research and Education Unit for Communication in Healthcare, Department of Cardiac Surgery, Ludwik Rydygier Collegium Medicum in Bydgoszcz Nicolaus Copernicus University in Torun, M. Curie Sklodowskiej St. 9, 85-094 Bydgoszcz, Poland; 2 Department of Oncology, Ludwik Rydygier Collegium Medicum in Bydgoszcz Nicolaus Copernicus University in Torun, Romanowskiej St. 2, 85-796 Bydgoszcz, Poland; 3 Departament de Enginyeria Química, Universitat Rovira i Virgili, Av. Països Catalans 26, Campus Sescelades, 43007 Tarragona, Spain; 4 Department of Cardiac Surgery, Ludwik Rydygier Collegium Medicum in Bydgoszcz Nicolaus Copernicus University in Torun, M. Curie Sklodowskiej St. 9, 85-094 Bydgoszcz, Poland; 5 Chemical Technologies Unit, Eurecat, Centre Tecnològic de Catalunya, Marcellí Domingo s/n, 43007 Tarragona, Spain; 6 Department of Tissue Engineering Chair of Urology and Andrology, Ludwik Rydygier Collegium Medicum in Bydgoszcz Nicolaus Copernicus University in Torun, Karlowicza St. 24, 85-092 Bydgoszcz, Poland

**Keywords:** COVID-19, Faith, Spirituality, Pandemic

## Abstract

The COVID-19 pandemic has impacted religion and faith in different ways. Numerous restrictions have been implemented worldwide. Believers are in conflict with authorities’ warnings that gatherings must be limited to combat the spread of the virus. Religion has always played a role of the balm for the soul, and the regular religious participation is associated with better emotional health outcomes. In our study, we examined whether the exposure to COVID-19 enhances the faith. The instrument used was a survey verifying the power of spirituality in the face of the coronavirus pandemic.

## Introduction

As the number of coronavirus confirmed cases approaches 845,000 in 202 countries and territories around the world (more than 41,000 deaths has been reported so far)^1^ and since scientists and politicians struggle to agree a response to the economic, social and health crisis due to the pandemic, many people are turning toward faith. The inspiration for this publication was strong words of the Polish Deputy Prime Minister who said that “Churches are like hospitals for the soul”. But is what is good for the soul always good for the body? Since the disease is a novelty to us, at first no protocols had really existed to fight it. It had not been subject to strict control, and referring to the data, Italy has become a hot spot of Europe with the largest number of people infected with Covid-19 after the virus’s expansion in China. The coronavirus outbreak in Europe has become a game changer for surveillance.

Over 98% of Poles are Christians, but only about 82% consider themselves to be actively practicing their religion. The rest reveals attending church services for a sense of duty or willingness to pass the tradition to their children. Modern European society is delineated by indifference to religious institutions or ideology and the concept of God functions as a force majeure, fate or destiny. However, in the face of illness and suffering, a significant change of attitude is observed, as evidenced by numerous testimonies of Italian doctors. Religious creeds and beliefs not only allow us to understand but they also influence the meaning of many events occurring in everyone’s life. With this approach, faith or broadly understood spirituality is a force that helps to overcome mental crisis as well as facilitate adaptation to the disease or the restrictions resulting from it.

In light of pandemic of COVID-19, most people are much more open to faith as well as prayer. The virus is going to be spreading rapidly and causing enormous crisis in all societies. In the current pandemic, engagement with religious practices gives us control over the situation, helps to make it understandable and what is most important gives us hope.

Taking the above into consideration, our study was aimed to verify the power of faith in the face of COVID-19 threat.

## Materials and Methods

### Study Design

The study group consisted of 324 respondents, and the survey was conducted among Polish society from March 13 to 16, 2020. The dates are of significance as it was the period when the Covid-19 fear has started to spread nationwide. The first case of infection with Covid-19 in Poland was reported on March 4, 2020. Since March 14, 2020, an epidemic emergency has been in force, and on March 15, 2020 a sanitary cordon was implemented around the Polish borders, significantly limiting border traffic. The survey was distributed among the general population via Google docs using popular mobile messengers and other social media channels.

The tool measured ten dimensions: sex, age, education, place of residence, faith, the essence of faith in life, the practice of prayer, the importance of faith/spirituality in connection with coronavirus danger, strengthening of the faith/spirituality in connection with the increasing coronavirus risk, the belief that faith/spirituality will increase the sense of security in the times of the pandemic.

### Data Collection Procedures

Data collection was carried out online and the designed tool used different scales, depending on questions. In order to gather socio-demographic data, we created our own part of the survey. For assessing the essence and the practice of prayer, we used 5-point Likert scale. For assessing the importance of faith/spirituality and its strengthening and influence, we used a yes/no questionnaire.

### Data Management and Analysis

Comparisons between variables in the described subgroups of men and women were evaluated using Student’s *t* test.

### Ethical Considerations

All procedures performed in the study involving human participants were in accordance with the ethical standards of the Institutional Research Committee (*Bioethical Committee of Ludwik Rydygier Collegium Medicum in Bydgoszcz, Nicolaus Copernicus University in Torun*). The survey was anonymous, conducted electronically, and did not require providing any personal data.

## Results

The study group consisted of 327 respondents (three respondents were excluded due to incomplete responses), yielding the final study population of 324 (*N* = 324). The study population is presented in Table 1. Of the 327 subjects surveyed, 50.6% were men and 49.4% were women. The largest group of respondents (54%) is situated in the age range of 21–35 years of age. The majority of the study group completed a secondary level of education (37.3%) or have college or university degree (52.2%). Almost half of the respondents declared living in cities of over 250,000 inhabitants (42.9%).

**Table 1:** Study population

Characteristic	% or *N* (%)
Sex	%
Female	49.4%
Male	50.6%
Age	*N* (%)
< 20	48 (14.8)
21–35	175 (54%)
36–50	63 (19.4%)
51–65	16 (4.9%)
66–80	18 (5.6%)
> 80	4 (1.2%)
Education	*N* (%)
Primary	5 (1.5%)
Junior high	21 (6.5%)
Vocational	8 (2.5%)
Upper high	121 (37.3%)
Tertiary	169 (52.2%)
Geographic data	N (%)
Village	60 (18.5%)
Town < 50,000 inhabitants	62 (19.1%)
City < 10,000 inhabitants	26 (8%)
City < 250,000 inhabitants	37 (11.4%)
City > 250,000 inhabitants	139 (42.9%)

With regards to the faith, among the vast majority of participants declared to be Catholics (91.7%, *N* = 297; 52.5% between the ages of 21 and 35; 72.4% < 50), within this group there were 51.5% of men (*N* = 153) and 48.5% of women (*N* = 144). Among those Catholic respondents who stated that faith plays a very important role in their lives, 57.9% were men and 42.1% were women. The instrument used in this part of the survey was the 5-point Likert scale where 1 was very important, 2 important, 3 rather important, 4 less important, 5 not important. Over half of the Catholic respondents disclosed that faith played a very important role in their lives (55.2%, *N* = 297; level 1 Likert scale); the largest group consisted of young people in the age range of 21-35 (62.2%). In comparison with the total number of participants (*N* = 324), level 1 Likert scale was declared by 51.5% (*N* = 167) and level 2 L.s. by 24,1% (*N* = 78).

We were also interested in examining the act of prayer practicing by the Catholic respondents. The obtained results revealed that 34,3% of the participants practices their religion very often (*N* = 297; 31.8% in *N* = 324; level 1 L.s.). Within this group, 44.1% were women and 55.8% were men. Almost 60% of the aforementioned group consisted of people between the ages of 21–35 (59.8%; 69.6% < 50).

For assessing the faith/spirituality importance and the strengthening and protective influence of faith/spirituality, we designed a yes/no questionnaire. The three queries of our interest were referenced to 1) the importance of faith/spirituality in connection with coronavirus danger, 2) strengthening of the faith/spirituality in connection with the increasing coronavirus risk and 3) the belief that faith/spirituality will increase the sense of security in the times of the pandemic. 67.6% of the respondents declared that faith/spirituality was important to them in the face of Covid-19 pandemic (*N* = 219); 72% of Catholics gave a positive answer to this query, and within this group there were 42.5% of women and 57.5% of men; the largest group consisted of young people in the age range of 21–35 (57%), respondents in the age range of 36–50: 12.14%, < 50: 69.16%, > 50: 30.84%. However, for the majority of the total number of the respondents, the religious involvement did not strengthen due to the risk of the infection (75.3%, *N* = 244; within the Catholic respondents 25,9% stated strengthening their faith). The outcomes obtained in the last section of the survey with regards to the belief in the protective power of faith and the increased sense of security over 40% responses revealed trust in such capability (40.7%, *N* = 132), compared to the Catholic respondents of whom 43.8% (*N* = 297) declared the belief in the protective power of faith (within this group 43.1% were women and 56.9% were men; 76% < 50).

In the aspect of education, the outcomes are worth further discussion. A crucial role of faith in life was declared by 72.05% (*N* = 297) of the Catholic respondents, including 45.79% of respondents who have college or university degree and 42.06% of respondents who completed a secondary level of education (87.85% in total). The majority of respondents did not declare that their faith had strengthened in the face of the Covid-19 pandemic threat (74.07% of “no” responses); within this group, 89.55% respondents completed a secondary level of education or have college or university degree. The last question of the survey investigated the increase of the respondents’ sense of security and their belief in the protective power of faith in the times of the pandemic. As many as 86.92% of respondents who completed a secondary level of education or have college or university degree stated a “yes” response.

## Discussion

Spirituality in the context of healthcare is a relatively new area yet becoming increasingly important. In the recent years, research has shown that religious beliefs and practices are associated with various health aspects, such as ability to cope with the disease, recovery after hospitalization and a positive attitude in a difficult situation, including health (Albers et al. 2010; Puchalski et al. 2009; Phelps et al. 2009). Therefore, the importance of spirituality in clinical practice has been highlited (Best et al. 2015). In general terms, spirituality is most often defined as the search for a “higher sense” with regards to religion or belief in God (Mishra et al. 2017). Most societies or people referred to as “Westerners” have successfully learned to resort to suffering and moral dilemmas. The convenience and comfort of life most often causes a lack of reflection as well as pushing away disturbing thoughts. The emergence of the Covid-19 pandemic has caused distinct human responses and reactions, has strengthened us and made us aware of the fragility of our human existence. We have been taught a lesson in humility, but we are also accompanied by feelings of powerlessness and fear.

In our survey in the face of the growing coronavirus pandemic, we were interested in the role of faith/spirituality in the general population of Poles, especially since the situation in Poland was not very serious at the time of our research study.

The analysis of the conducted survey, with 324 participants, showed that mainly young people, both women and men, dominated. In the group of young people between the ages of 21 and 35, the essence of faith was of a great importance and was declared to be accompanied by the frequent practice of prayer. In Poland, there has been a discussion of a growing crisis of faith in the younger generation and their lack of attachment to church traditions. This survey group makes an extremely interesting case for further analysis because it has never experienced such a social disaster before. Our research proves that women more often declare strengthening their faith/spirituality in the face of the coronavirus hazard. Also worldwide research indicates that women participate in religious life more often, pray more or feel a greater presence of God in everyday life (Forlenza and Vallada 2018; Peteet et al. 2019; Li et al. 2016). To a large extent, this is probably due to psychological differences between men and women, also in the metaphysical sphere. At the same time, within this study group, young women believe that faith will protect them from the coronavirus infection (64%). Perhaps this is related to the image of God as a good and merciful father who will be able to save us from all evil and suffering. In general, the development of faith is also observed in the group of the elderly, which may be caused by the awareness of the inevitability of death (Harrington 2016; Hodge et al. 2011). The coronavirus pandemic, however, also caused fear of death in younger people, which is compounded by the world media reports about the deaths of young people who are not burdened with additional diseases. Such anxiety more often accompanies women, which is also reported and proved in our study. Women appreciate family values very much, care for the needs of their loved ones and often entrust their fate to divine protection (Simon 1995). Emphasized should also be the fact that completing the survey resembles a declaration, and men are less willing to admit their beliefs or religious values. Hence, in a sense, there may be differences between sexes, also visible in the presented research study.

To conclude, when being exposed to a threat we use various strategies of survival, faith being one of them, which allows us to keep hope as well as feel sense of security. The current worldwide situation can bring people together, also through joint prayer.

## Conclusions

Modern societies, prior to the Covid-19 pandemic, focused mainly on the body and well-being, largely excluding spirituality and thus narrowing human desires only to the physical sphere. A man of body and emotions dominated over a man of spirit. Our research conducted as part of the survey indicates that people experiencing fear, suffering or illness often experience a “spiritual renewal.” Perhaps a new “generation of coronavirus” is being shaped, in which the development of spirituality will create the mature attitude based on truth and freedom.

## Footnotes

1 Last updated: March 31, 2020 acc. to https://www.worldometers.info/coronavirus/.
